# Ricardo Velluti, a Pioneer in Latin American Sleep Research

**DOI:** 10.5935/1984-0063.20220049

**Published:** 2022

**Authors:** Monica Levy Andersen, Sergio Tufik

**Affiliations:** Departamento de Psicobiologia, Universidade Federal de São Paulo - São Paulo - Brazil

The Latin American sleep medicine and research community received with great sadness the news of Ricardo Velluti’s death, who passed away on June 18^th^, 2022. Dr. Velluti was a prominent Uruguayan neuroscientist and a pioneer in the study of sleep in Uruguay and Latin America. He graduated from the Faculty of Medicine of the Universidad de la República - Montevideo, serving as a professor in the Department of Physiology since 1985. From this department he published most of his seminal works on the interplay between the sleep-wake cycle and sensory neurophysiology, mostly auditory system. Later in life, he became an Honorary Professor at the University Latin American Center for Human Economics (CLAEH) in Punta del Este, Uruguay, where he continued working on sleep neurobiology.

Dr. Velluti began working with sleep research on the decades of 1960 to 1980, a time in which sleep research was more related to basic physiology than to medical practice. Early in his scientific career, he become interested in two topics related to neurophysiology, which were initially conducted as parallel research lines. The first was related to sleep neurobiology, especially focused on brain pO_2_ control during sleep and wakefulness^1^-^4^, while the second was devoted to auditory neurophysiology^5^-^7^. Eventually, he merged both topics into a single one and this combination brought a deep insight into the mechanisms of information processing during the different stages of the sleep-wake cycle^8^-^13^.

He kept working on until recently and his works contributed greatly to the development of basic research on the effects of sleep on sensory processing, providing important clinical insights to the field. The research carried out in Velluti’s laboratory concerned the influence of acoustic stimulation on sleep behavior and the mechanisms of neuronal processing of acoustic information along the complex auditory pathways of the central nervous system during the wake-sleep cycle.

Altogether, Dr. Velluti published more than 70 articles. We have had the opportunity to publish a couple of them at *Sleep Science*, with highlights to a brilliant review about the participation of neurophysiological sensory functions in active sleep processes^14^-^16^. He was the editor and author of two fundamental books: *The Auditory System in Sleep^17^* and *The Physiologic Nature of Sleep^18^*, together with another forefather of sleep medicine research, Dr. Pier Luigi Parmeggiani.

Along all these studies and publications, Dr. Velluti has worked with many other renowned Uruguayan sleep researchers, including Dr. Jaime Monti, Dr. José Luis Peña, Dr. Pablo Torterolo, and Dr. Marisa Pedemonte, being also responsible for training several researchers in the area. With Marisa the relationship went beyond work, and we can surely say they were the most lovely couple in Latin American sleep research. They married, had children, become lifelong partners and lived more than 35 years together.

Dr. Velluti also played a significant role in the management of science in his home country, participating as a member of the *Comisión Nacional de Investigación Ciencia y Tecnología* (CONICET), being the founder of the first *Sociedad Uruguaya de Investigación en Sueño*.

We have had the pleasure to spent nice moments with Dr. Velluti, in congresses and meetings across Latin America and beyond. In 2018, we were pleased to share the offering of the “Velluti Prize” for basic research and the “Tufik Prize” for clinical research at the Congress of the Latin American Federation of Sleep Societies (FLASS) in Punta del Este, Uruguay.

The sleep research community will miss this exceptional researcher. Ricardo Velluti was a brilliant scientist and stood out with excellence in our continent. His work has placed him at the forefront of the approach of auditory processing during sleep and wakefulness. We are sure his legacy will continue and his works will remain inspiring generations of Latin American researchers.
